# Supplementary material for: Genetics-mediated regulation of intestinal gene expression on microbiome contributes to human disease heritability
Source: Mol Syst Biol. 2025 Dec 5;22(3):369–94. doi: 10.1038/s44320-025-00173-7 (PMC12953866; doi:10.1038/s44320-025-00173-7)
Supplement: Supplementary file 21 — Expanded View Figures [file 44320_2025_173_MOESM21_ESM.pdf]

Expanded View Figures

Figure EV1. Genetic regulatory maps between gene expression and microbiome.

(**A**) Manhattan plot illustrating the significant gene expression-to-microbiome regulatory pairs in the sigmoid colon and ileum. Each dot represents the gene, with the *x* axis indicating the position and the *y* axis means the $-\log_{10}$ *P* value. (**B**) Effect size consistency the MR regulatory pairs identified based on GTEx (discovery) versus CEDAR (replication) datasets. Pearson correlation coefficient and the corresponding *P* value were calculated. (**C**) Distribution of the significant gene-to-microbiome regulatory pairs in each genus across three tissues. The box's central line indicates the median. The bounds of the box represent the 25th and 75th percentiles (interquartile range, IQR). The whiskers extend to the most extreme data points that are within 1.5 × IQR from the box. The value of n shown in each *x* axis label represents the number of gene-to-microbiome regulatory pairs used for that genus. (**D**) The effect sizes of the gene-to-microbiome regulation of the top 20 genera in the sigmoid colon and ileum. The *y* axis indicates the absolute effect size, and the *x* axis indicates the top 20 genera with the strongest overall regulatory magnitude. The box's central line indicates the median. The bounds of the box represent the 25th and 75th percentiles (interquartile range, IQR). The whiskers extend to the most extreme data points that are within 1.5 × IQR from the box. The value of n shown in each *x* axis label represents the number of gene-to-microbiome regulatory pairs used for that genus. (**E**) Box plot indicating the functional annotation of the genes from the regulatory MR pairs on chromosome 12 in three tissues. (**F**) Number of MR-based microbiome-to-gene pairs, colocalization pairs across three tissues. (**G**) Number of MR-based microbiome-to-methylation pairs, colocalization pairs in the transverse colon. Source data are available online for this figure.

 © The Author(s)

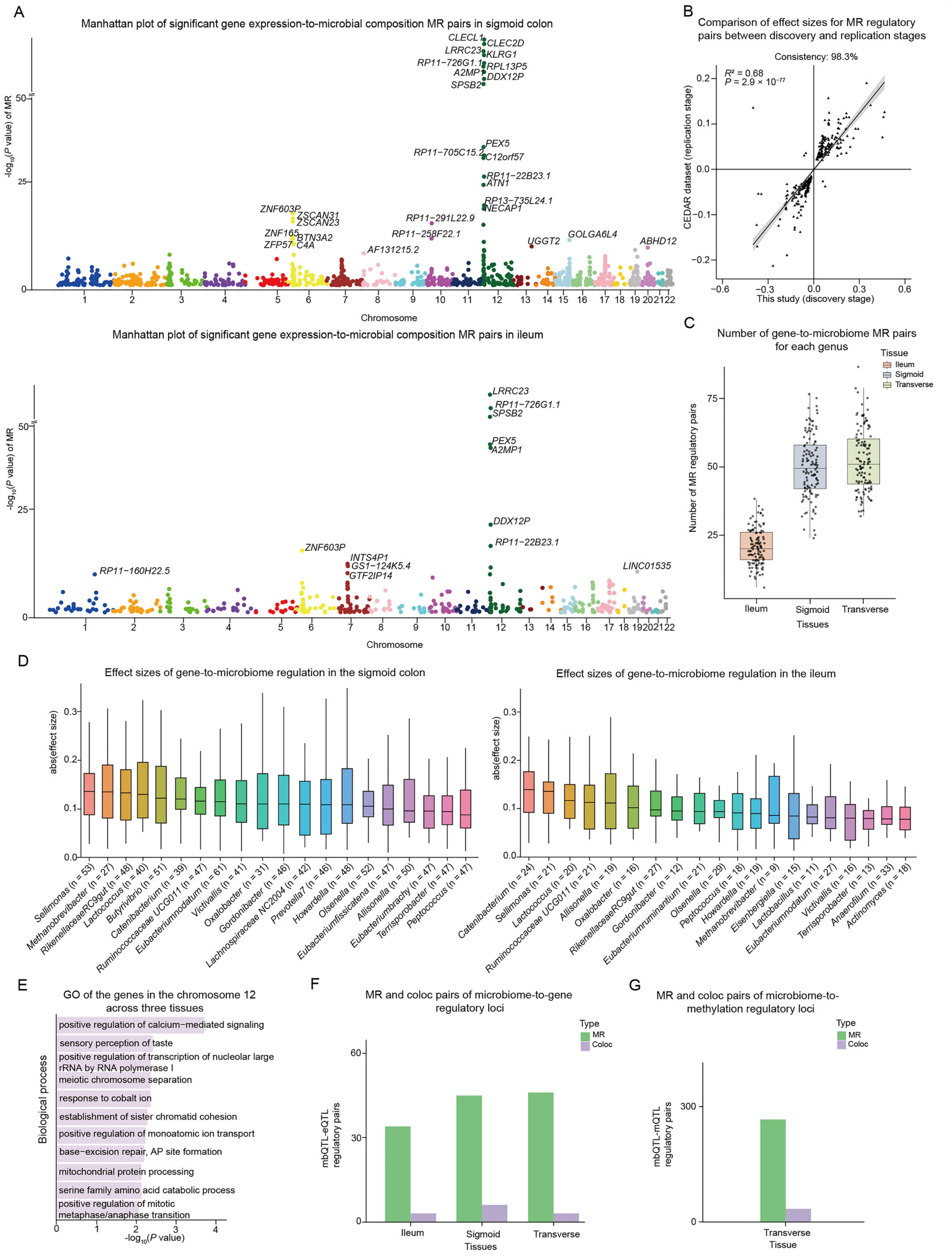
A
Manhattan plot of significant gene expression-to-microbial composition MR pairs in sigmoid colon
-log10(P value) of MR
Chromosome
CLECL1
CLEC2D
LRRC23
KLRG1
RP11−726G1.1
RPL13P5
A2MP1
DDX12P
SPSB2
PEX5
RP11−705C15.2
C12orf57
RP11−22B23.1
ATN1
RP13−735L24.1
NECAP1
ZNF603P
ZSCAN31
ZSCAN23
RP11−291L22.9
ZNF165
BTN3A2
RP11−258F22.1
ZFP57
C4A
AF131215.2
UGGT2
GOLGA6L4
ABHD12
Manhattan plot of significant gene expression-to-microbial composition MR pairs in ileum
LRRC23
RP11−726G1.1
SPSB2
PEX5
A2MP1
DDX12P
RP11−22B23.1
ZNF603P
INTS4P1
GS1−124K5.4
GTF2IP14
RP11−160H22.5
LINC01535
B
Comparison of effect sizes for MR regulatory pairs between discovery and replication stages
Consistency: 98.3%
R² = 0.68
P = 2.9 × 10⁻⁷⁷
CEDAR dataset (replication stage)
This study (discovery stage)
C
Number of gene-to-microbiome MR pairs for each genus
Tissue
Ileum
Sigmoid
Transverse
Number of MR regulatory pairs
Tissues
D
Effect sizes of gene-to-microbiome regulation in the sigmoid colon
abs(effect size)
Sellimonas (n = 53)
Methanobrevibacter (n = 27)
RikenellaceaeRC9gut (n = 48)
Lactococcus (n = 40)
Butyrivibrio (n = 51)
Catenibacterium (n = 39)
Ruminococcaceae UCG011 (n = 47)
Eubacteriumnodatum (n = 61)
Victivallis (n = 41)
Oxalobacter (n = 31)
Gordonibacter (n = 46)
Lachnospiraceae NC2004 (n = 42)
Prevotella7 (n = 46)
Howardella (n = 48)
Olsenella (n = 52)
Eubacteriumfissicatena (n = 47)
Allisonella (n = 50)
Eubacteriumbrachy (n = 47)
Terrisporobacter (n = 47)
Peptococcus (n = 47)
Effect sizes of gene-to-microbiome regulation in the ileum
Catenibacterium (n = 24)
Sellimonas (n = 21)
Lactococcus (n = 20)
Ruminococcaceae UCG011 (n = 21)
Allisonella (n = 19)
Oxalobacter (n = 16)
RikenellaceaeRC9gut (n = 27)
Gordonibacter (n = 12)
Eubacteriumruminantium (n = 21)
Olsenella (n = 29)
Peptococcus (n = 18)
Howardella (n = 19)
Methanobrevibacter (n = 9)
Eisenbergiella (n = 15)
Lactobacillus (n = 11)
Eubacteriumnodatum (n = 27)
Victivallis (n = 16)
Terrisporobacter (n = 13)
Anaerofilum (n = 33)
Actinomyces (n = 18)
E
GO of the genes in the chromosome 12 across three tissues
Biological process
positive regulation of calcium−mediated signaling
sensory perception of taste
positive regulation of transcription of nucleolar large rRNA by RNA polymerase I
meiotic chromosome separation
response to cobalt ion
establishment of sister chromatid cohesion
positive regulation of monoatomic ion transport
base−excision repair, AP site formation
mitochondrial protein processing
serine family amino acid catabolic process
positive regulation of mitotic metaphase/anaphase transition
-log10(P value)
F
MR and coloc pairs of microbiome-to-gene regulatory loci
Type
MR
Coloc
mbQTL-eQTL regulatory pairs
Ileum
Sigmoid
Transverse
Tissues
G
MR and coloc pairs of microbiome-to-methylation regulatory loci
Type
MR
Coloc
mbQTL-mQTL regulatory pairs
Transverse
Tissue

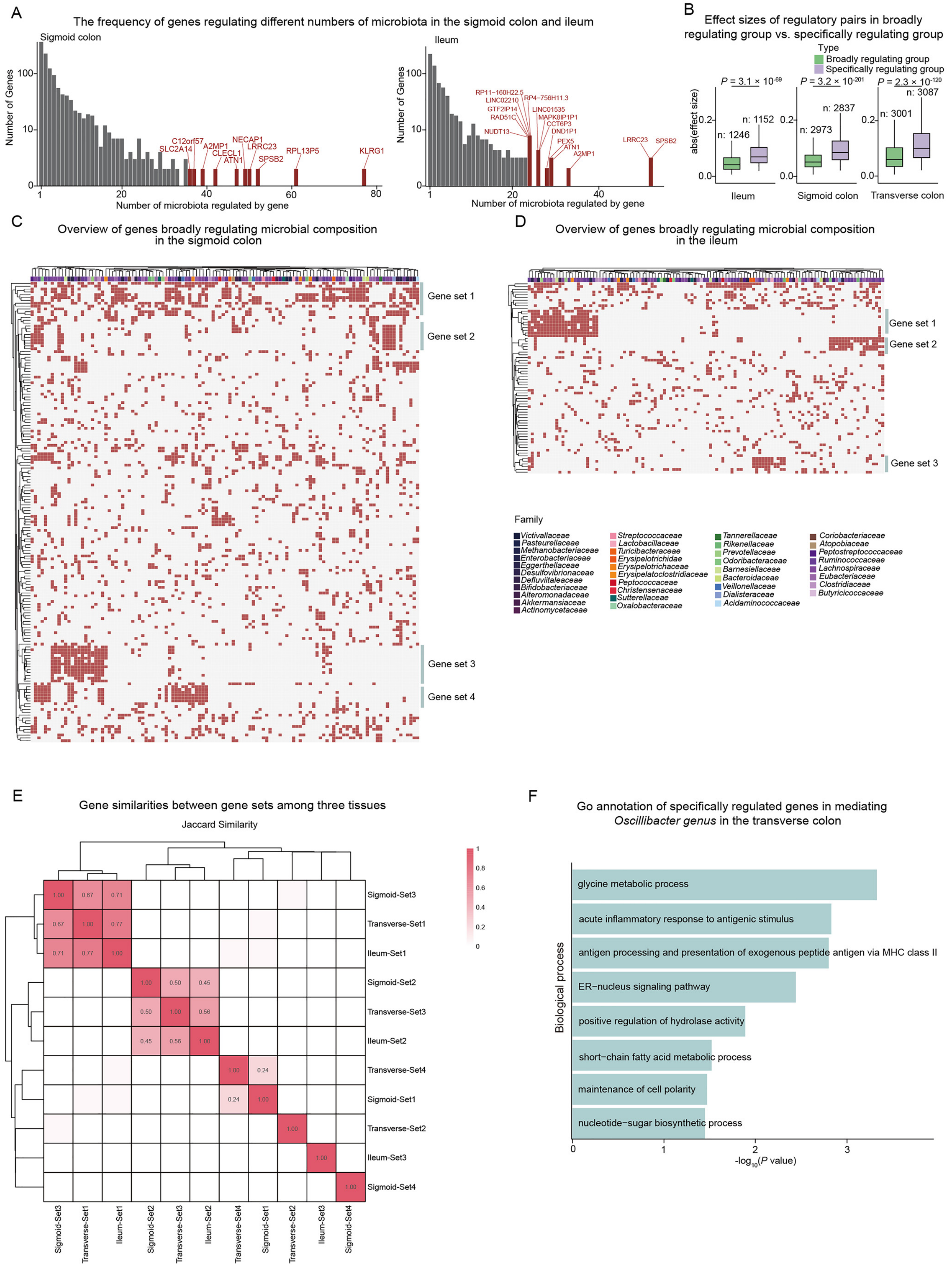
A
The frequency of genes regulating different numbers of microbiota in the sigmoid colon and ileum
Sigmoid colon
Number of Genes
Number of microbiota regulated by gene
C12orf57
SLC2A14
A2MP1
CLECL1
ATN1
NECAP1
LRRC23
SPSB2
RPL13P5
KLRG1
Ileum
RP11−160H22.5
LINC02210
GTF2IP14
RAD51C
NUDT13
RP4−756H11.3
LINC01535
MAPK8IP1P1
CCT6P3
DND1P1
PEX5
ATN1
A2MP1
LRRC23
SPSB2
Number of microbiota regulated by gene
B
Effect sizes of regulatory pairs in broadly regulating group vs. specifically regulating group
Type
Broadly regulating group
Specifically regulating group
P = 3.1 × 10-69
P = 3.2 × 10-201
P = 2.3 × 10-120
abs(effect size)
n: 1246
n: 1152
n: 2973
n: 2837
n: 3001
n: 3087
Ileum
Sigmoid colon
Transverse colon
C
Overview of genes broadly regulating microbial composition in the sigmoid colon
Gene set 1
Gene set 2
Gene set 3
Gene set 4
D
Overview of genes broadly regulating microbial composition in the ileum
Gene set 1
Gene set 2
Gene set 3
Family
Victivallaceae
Pasteurellaceae
Methanobacteriaceae
Enterobacteriaceae
Eggerthellaceae
Desulfovibrionaceae
Defluviitaleaceae
Bifidobacteriaceae
Alteromonadaceae
Akkermansiaceae
Actinomycetaceae
Streptococcaceae
Lactobacillaceae
Turicibacteraceae
Erysipelotrichidae
Erysipelotrichaceae
Erysipelatoclostridiaceae
Peptococcaceae
Christensenaceae
Sutterellaceae
Oxalobacteraceae
Tannerellaceae
Rikenellaceae
Prevotellaceae
Odoribacteraceae
Barnesiellaceae
Bacteroidaceae
Veillonellaceae
Dialisteraceae
Acidaminococcaceae
Coriobacteriaceae
Atopobiaceae
Peptostreptococcaceae
Ruminococcaceae
Lachnospiraceae
Eubacteriaceae
Clostridiaceae
Butyricicoccaceae
E
Gene similarities between gene sets among three tissues
Jaccard Similarity
Sigmoid-Set3
Transverse-Set1
Ileum-Set1
Sigmoid-Set2
Transverse-Set3
Ileum-Set2
Transverse-Set4
Sigmoid-Set1
Transverse-Set2
Ileum-Set3
Sigmoid-Set4
F
Go annotation of specifically regulated genes in mediating Oscillibacter genus in the transverse colon
Biological process
glycine metabolic process
acute inflammatory response to antigenic stimulus
antigen processing and presentation of exogenous peptide antigen via MHC class II
ER−nucleus signaling pathway
positive regulation of hydrolase activity
short−chain fatty acid metabolic process
maintenance of cell polarity
nucleotide−sugar biosynthetic process
-log10(P value)

 © The Author(s)

Figure EV2. Functional enrichments of gene-to-microbiome regulatory loci.

(**A**) Bar plot indicating the distribution of the number of genes based on the number of microbiota they regulated in the sigmoid colon and ileum. Genes regulating more than 50 microbiota genera were labeled. (**B**) Comparison of the overall effect sizes of gene-to-microbiome pairs between broadly regulating groups and specifically regulating groups in the three tissues. The box's central line indicates the median. The bounds of the box represent the 25th and 75th percentiles (interquartile range, IQR). The whiskers extend to the most extreme data points that are within 1.5 × IQR from the box. The value of n shown above each box represents the number of gene-to-microbiome regulatory pairs in each group. (**C**) Heatmap showing the genes that broadly regulated microbiota in the sigmoid colon, with five and three gene sets formed via hierarchical clustering. (**D**) Heatmap showing the genes that broadly regulated microbiota in the ileum, with five and three gene sets formed via hierarchical clustering. (**E**) Heatmap indicating the similarity level (Jaccard similarity index) between gene sets across the three tissues. The Jaccard index was measured by the presence-absence gene matrix for three tissues. (**F**) An example of the GO enrichment for the genes that specifically regulated the *Oscillibacter* genus. Functional enrichments were calculated by using Fisher's exact test. Source data are available online for this figure.

© The Author(s)

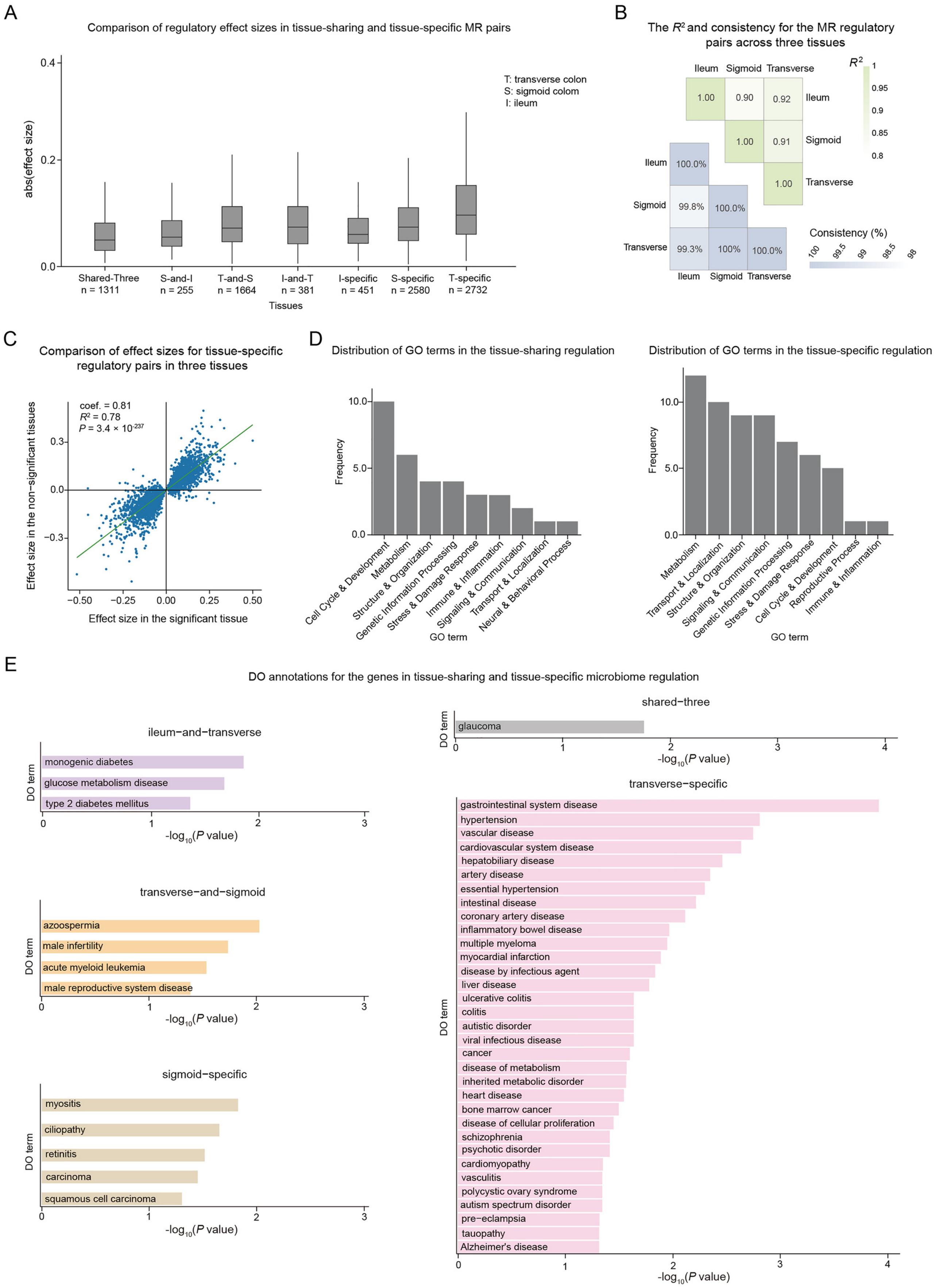
A
Comparison of regulatory effect sizes in tissue-sharing and tissue-specific MR pairs
abs(effect size)
T: transverse colon
S: sigmoid colom
I: ileum
Shared-Three n = 1311
S-and-I n = 255
T-and-S n = 1664
I-and-T n = 381
I-specific n = 451
S-specific n = 2580
T-specific n = 2732
Tissues
B
The R² and consistency for the MR regulatory pairs across three tissues
Ileum Sigmoid Transverse
1.00 0.90 0.92 Ileum
1.00 0.91 Sigmoid
1.00 Transverse
Ileum 100.0%
Sigmoid 99.8% 100.0%
Transverse 99.3% 100% 100.0%
Consistency (%)
C
Comparison of effect sizes for tissue-specific regulatory pairs in three tissues
coef. = 0.81
R² = 0.78
P = 3.4 × 10-237
Effect size in the non-significant tissues
Effect size in the significant tissue
D
Distribution of GO terms in the tissue-sharing regulation
Frequency
Cell Cycle & Development
Metabolism
Structure & Organization
Genetic Information Processing
Stress & Damage Response
Immune & Inflammation
Signaling & Communication
Transport & Localization
Neural & Behavioral Process
GO term
Distribution of GO terms in the tissue-specific regulation
Metabolism
Transport & Localization
Structure & Organization
Signaling & Communication
Genetic Information Processing
Stress & Damage Response
Cell Cycle & Development
Reproductive Process
Immune & Inflammation
GO term
E
DO annotations for the genes in tissue-sharing and tissue-specific microbiome regulation
ileum−and−transverse
monogenic diabetes
glucose metabolism disease
type 2 diabetes mellitus
transverse−and−sigmoid
azoospermia
male infertility
acute myeloid leukemia
male reproductive system disease
sigmoid−specific
myositis
ciliopathy
retinitis
carcinoma
squamous cell carcinoma
shared−three
glaucoma
transverse−specific
gastrointestinal system disease
hypertension
vascular disease
cardiovascular system disease
hepatobiliary disease
artery disease
essential hypertension
intestinal disease
coronary artery disease
inflammatory bowel disease
multiple myeloma
myocardial infarction
disease by infectious agent
liver disease
ulcerative colitis
colitis
autistic disorder
viral infectious disease
cancer
disease of metabolism
inherited metabolic disorder
heart disease
bone marrow cancer
disease of cellular proliferation
schizophrenia
psychotic disorder
cardiomyopathy
vasculitis
polycystic ovary syndrome
autism spectrum disorder
pre−eclampsia
tauopathy
Alzheimer's disease
DO term
-log10(P value)

 © The Author(s)

Figure EV3. Tissue specificity and sharing of the gene-to-microbiome regulation.

(**A**) Comparison of the effect sizes in the tissue-sharing and tissue-specific groups, with *x* axis illustrating the MR regulatory types, and *y* axis showing the absolute value of the effect size. The box's central line indicates the median. The bounds of the box represent the 25th and 75th percentiles (interquartile range, IQR). The whiskers extend to the most extreme data points that are within 1.5 × IQR from the box. The value of *n* shown beneath each *x* axis label represents the number of gene-to-microbiome regulatory pairs in each group. (**B**) Heatmap illustrating the R^2 (upper triangle) and consistency (lower triangle) for the regulatory pairs across three intestinal tissues. (**C**) Comparisons of the effect sizes for the tissue-specific regulatory pairs between the significant tissue versus other tissues. Regression coefficient, Pearson correlation coefficients, and the corresponding *P* values were calculated. (**D**) Bar plots indicating GO term distributions in the tissue-sharing and tissue-specific regulation. (**E**) Bar plots indicating the DO enrichment for the genes involved in the tissue-sharing and tissue-specific regulation categories. DO enrichments were calculated by using Fisher's exact test. Source data are available online for this figure.

© The Author(s)

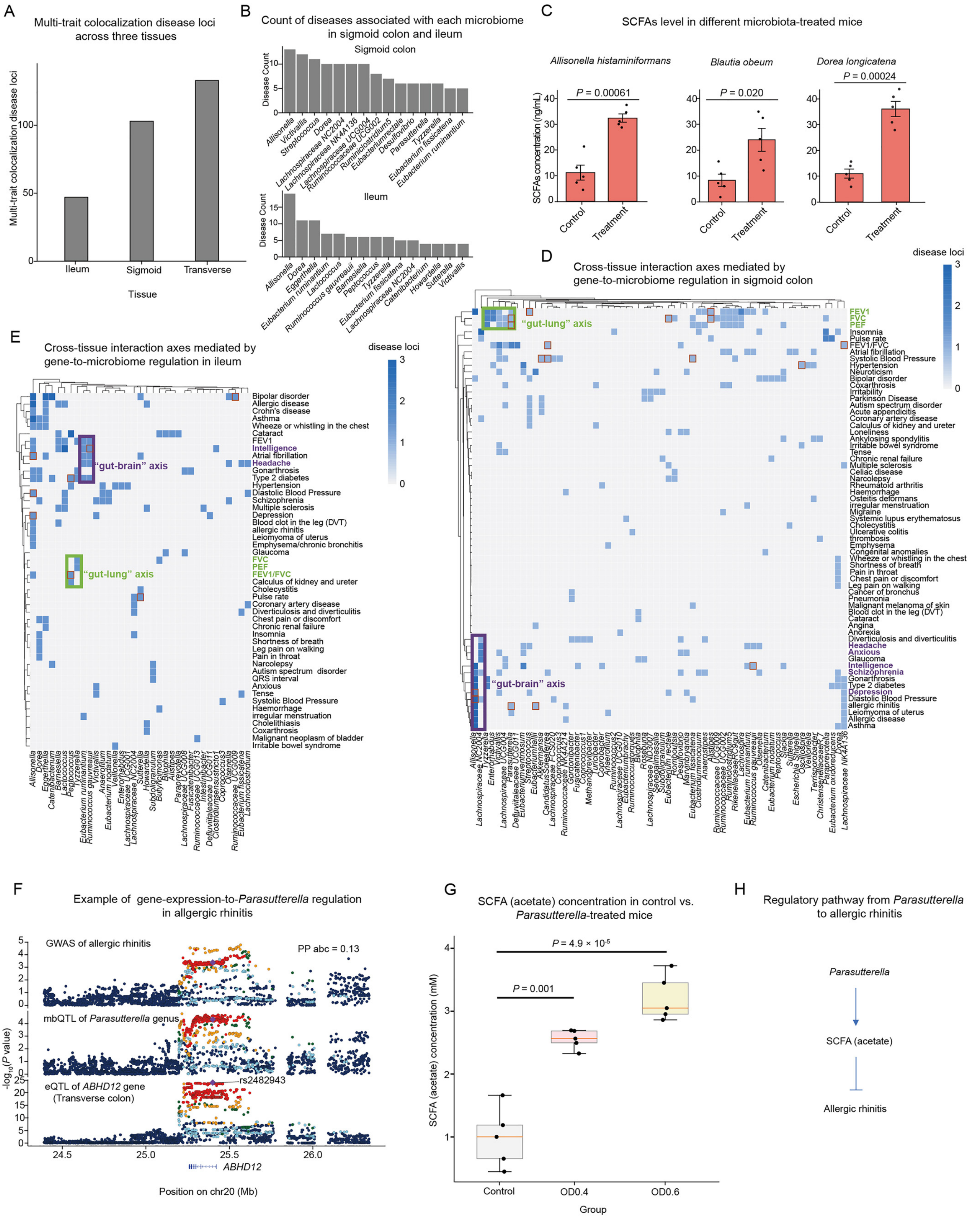
A
Multi-trait colocalization disease loci across three tissues
Multi-trait colocalization disease loci
Ileum
Sigmoid
Transverse
Tissue
B
Count of diseases associated with each microbiome in sigmoid colon and ileum
Sigmoid colon
Ileum
Disease Count
C
SCFAs level in different microbiota-treated mice
Allisonella histaminiformans
Blautia obeum
Dorea longicatena
P = 0.00061
P = 0.020
P = 0.00024
SCFAs concentration (ng/mL)
Control
Treatment
D
Cross-tissue interaction axes mediated by gene-to-microbiome regulation in sigmoid colon
disease loci
"gut-lung" axis
"gut-brain" axis
E
Cross-tissue interaction axes mediated by gene-to-microbiome regulation in ileum
disease loci
"gut-brain" axis
"gut-lung" axis
F
Example of gene-expression-to-Parasutterella regulation in allgergic rhinitis
GWAS of allergic rhinitis
PP abc = 0.13
mbQTL of Parasutterella genus
eQTL of ABHD12 gene (Transverse colon)
rs2482943
-log10(P value)
ABHD12
Position on chr20 (Mb)
G
SCFA (acetate) concentration in control vs. Parasutterella-treated mice
P = 4.9 × 10-5
P = 0.001
SCFA (acetate) concentration (mM)
Control
OD0.4
OD0.6
Group
H
Regulatory pathway from Parasutterella to allergic rhinitis
Parasutterella
SCFA (acetate)
Allergic rhinitis

 © The Author(s)

Figure EV4. Gene-to-microbiome regulation underlying genetic diseases.

(**A**) Bar plot indicating the number of multi-trait colocalization disease loci across three tissues. (**B**) Bar plots illustrating the number of genetic diseases and traits (*y* axis) associated with different microbiota (*x* axis) identified through multi-trait colocalization analysis in the sigmoid colon and ileum. (**C**) Experimental validations for the regulatory effects of different microbiota on (short-chain fatty acid) SCFA production. *P* value for each comparison was calculated by unpaired *t* test. Each group contained 5 replicates. Error bars indicate standard error of the mean. (**D**) Heat maps indicating the multi-trait colocalization between complex diseases (row) and the genera (column) involved in gene-to-microbiome regulation pairs in the sigmoid colon, with the color scale representing the number of loci. The red boxes represent the loci that were further supported by two-step MR analyses. (**E**) Heat maps indicating the multi-trait colocalization between complex diseases (row) and the genera (column) involved in gene-to-microbiome regulation pairs in the ileum, with the color scale representing the number of loci. The red boxes represent the loci that were further supported by two-step MR analyses. (**F**) LocusCompare visualization of the multi-trait localization across allergic rhinitis GWAS, *Parasutterella* genus mbQTL, and *ABHD12* eQTL in the transverse colon. The *y* axis shows log-changed *P* value of association tests in GWAS, mbQTLs, and eQTLs. The lead variant is indicated, and the correlation of the LD effect relative to the lead variant is represented by the color scale. (**G**) Box plot representing comparisons of the SCFA (acetate) concentration between control mice and mice treated with different concentrations of *Parasutterella*. The box's central line indicates the median. The bounds of the box represent the 25th and 75th percentiles (interquartile range, IQR). The whiskers extend to the most extreme data points that are within 1.5 × IQR from the box. *P* value for each comparison was calculated by unpaired *t* test. Each group contains 5 replicates. (**H**) Schematic illustrating the regulatory axis across *Parasutterella* abundance, SCFA level, and allergic rhinitis. Source data are available online for this figure.

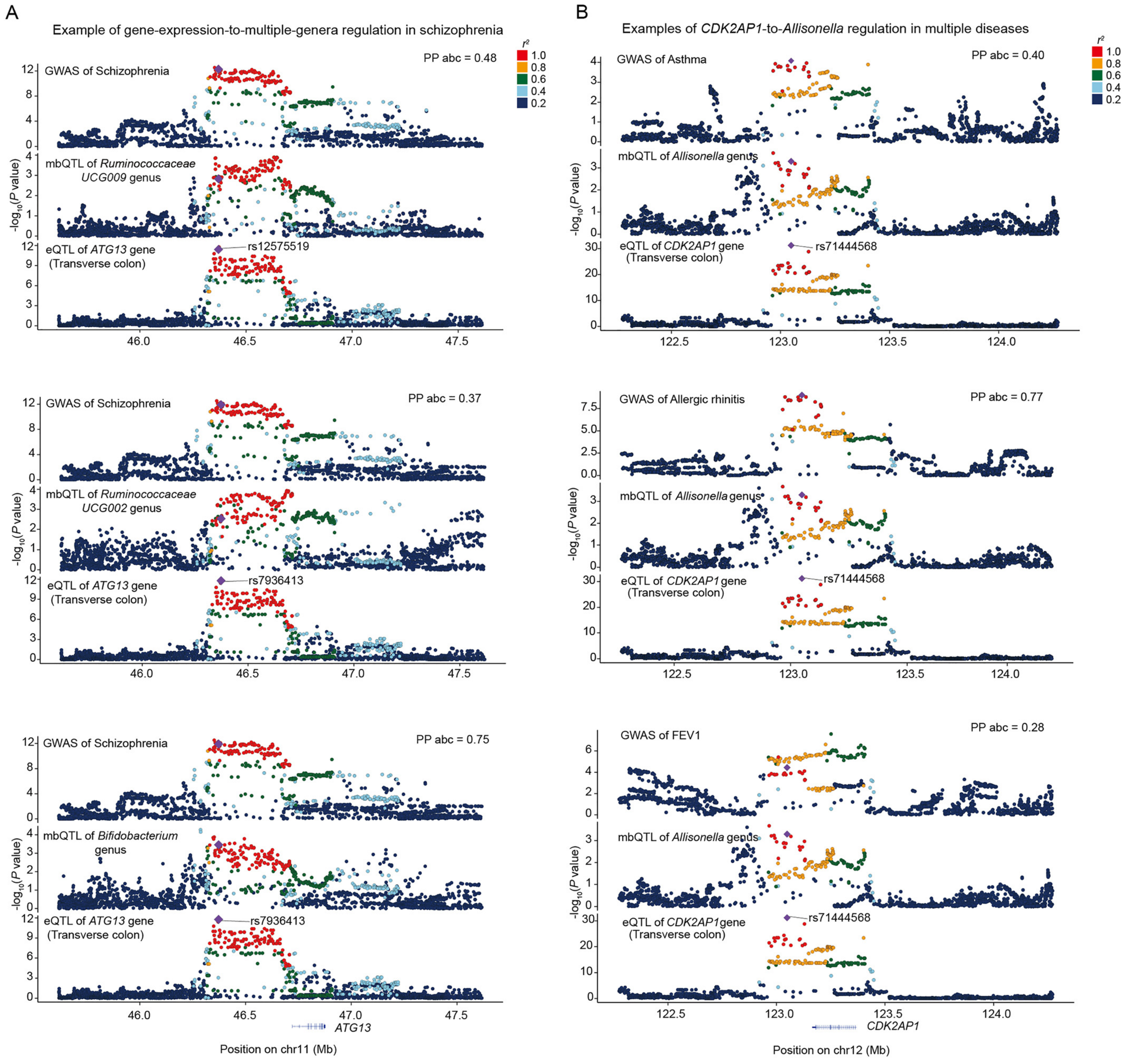

Figure EV5. Pleiotropic effect of gene-to-microbiome regulation on the diseases.

(**A**) LocusCompare plot of a pleiotropic disease locus mediated by various microbial genera, including *Ruminococcaceae UCG009* (top), *Ruminococcaceae UCG002* (middle), *and Bifidobacterium* (bottom), for their colocalization with schizophrenia GWAS and *ATG13* locus in the transverse colon. The lead variant and the posterior probability for each multi-trait colocalization are shown. (**B**) LocusCompare plots showing *CDK2AP1*-to-*Allisonella regulation* is associated with various diseases, including asthma, allergic rhinitis and FEV in the transverse colon, the lead variant and the posterior probability for each multi-trait colocalization are shown. Source data are available online for this figure.

 © The Author(s)
